# Infarct Timing and Predictors of Infarct-Free Survival in Patients with Aneurysmal Subarachnoid Hemorrhage

**DOI:** 10.3390/brainsci15101042

**Published:** 2025-09-25

**Authors:** Pikria Ketelauri, Meltem Gümüs, Aigerim Togyzbayeva, Hanah Hadice Karadachi, Anna Michel, Emad Mohajerani, Christoph Rieß, Thiemo Florin Dinger, Laurèl Rauschenbach, Marvin Darkwah Oppong, Yahya Ahmadipour, Philipp Dammann, Ulrich Sure, Ramazan Jabbarli

**Affiliations:** 1 Department of Neurosurgery and Spine Surgery, University Hospital Essen, Hufelandstrasse 55, 45147 Essen, Germany; meltem.guemues@uk-essen.de (M.G.); aigerim.togyzbayeva@uk-essen.de (A.T.); hanah.karadachi@uk-essen.de (H.H.K.); anna.michel@uk-essen.de (A.M.); emad.mohajerani@uk-essen.de (E.M.); christoph.riess@uk-essen.de (C.R.); thiemo-florin.dinger@uk-essen.de (T.F.D.); laurel.rauschenbach@uk-essen.de (L.R.); marvin.darkwahoppong@uk-essen.de (M.D.O.); yahya.ahmadipour@uk-essen.de (Y.A.); philipp.dammann@uk-essen.de (P.D.); ulrich.sure@uk-essen.de (U.S.); ramazan.jabbarli@uk-essen.de (R.J.); 2 Hospital for Sick Children, University of Toronto, 170 Elizabeth St., Toronto, ON M5G 1E8, Canada

**Keywords:** aneurysmal subarachnoid hemorrhage, cerebral infarction, infarct timing, DCI infarct, predictors of infarct timing

## Abstract

Background/Objectives: Cerebral infarction significantly worsens outcomes after aneurysmal subarachnoid hemorrhage (SAH). This retrospective study analyzed early predictors of infarct-free survival and the impact of infarct timing on clinical outcomes. Methods: We reviewed 988 consecutive SAH patients treated from 2003 to 2016, all with follow-up CT scans. Baseline clinical and SAH characteristics were recorded to identify predictors of infarct-free survival and assess the relationship between infarct timing and outcomes. Results: Cerebral infarctions occurred in 475 patients (48.1%) at a median of 3.4 days post-SAH; 70.9% happened within the first week. Earlier infarctions were associated with higher in-hospital mortality (odds ratio [OR] = 0.91 per day increase, *p* < 0.0001) and poor 6-month outcomes (modified Rankin Scale > 3; OR = 0.96 per day increase, *p* = 0.012), especially within 48 h. Independent predictors of infarct-free survival included poor initial condition (WFNS ≥ 4, adjusted hazard ratio [aHR] = 1.82, *p* < 0.0001), intraventricular hemorrhage (aHR = 1.25, *p* = 0.041), aneurysm rebleeding (aHR = 1.76, *p* < 0.0001), acute hydrocephalus (aHR = 1.38, *p* = 0.020), and daily aspirin intake (aHR = 0.68, *p* = 0.002). The number of baseline risk factors (0–5) strongly influenced both infarction likelihood and timing (*p* < 0.0001). Conclusions: Cerebral infarctions predominantly occur within the first week after SAH, with earlier infarctions having a more severe impact on outcomes. Initial risk factor-adapted SAH management may improve functional outcomes.

## 1. Introduction

Aneurysmal subarachnoid hemorrhage (SAH) is a severe form of intracranial bleeding that frequently leads to significant health complications and carries a high risk of mortality and long-term morbidity. Despite substantial advancements in diagnosis and treatment, SAH still carries an overall mortality rate of approximately 35% [1,2,3], with around 20% of survivors experiencing considerable morbidity [3,4]. One of the most severe complications is cerebral infarction, which can profoundly impact overall survival and neurological recovery [3,5,6,7]. Even after rehabilitation, many individuals continue to face significant functional impairments, largely dictated by the severity of their initial clinical and neurological status [3,8].

Cerebral infarction is a frequent complication in patients with SAH, with a reported prevalence ranging from 13% to 65% [9,10,11,12]. The causes of this event may be attributed to various factors, including early brain injury, aneurysm rebleeding, complications during aneurysm treatment, increased intracranial pressure (ICP), and cerebral vasospasm [9,13,14,15,16,17]. Current research primarily differentiates between early and late infarctions; however, the precise temporal patterns of cerebral infarct occurrence following SAH remain poorly understood. It is not yet entirely clear which risk factors influence the timing of infarction, and which early predictors are associated with infarct-free survival. A comprehensive understanding of the temporal dynamics of cerebral infarction, its underlying mechanisms, and its impact on patient outcomes is crucial for optimizing clinical management and improving prognoses in SAH patients.

In this large retrospective study, we aimed to expand the current evidence regarding post-SAH infarct timing. We analyzed the baseline patients and SAH characteristics, as well as factors that were already known at the time of aneurysm treatment, and investigated their influence on infarct-free survival. Furthermore, we investigated the clinical impact of the timing of infarct onset on short- and long-term SAH outcome.

## 2. Materials and Methods

### 2.1. Patient Population

Consecutive cases with aneurysmal SAH treated at our institution between January 2003 and June 2016 who had at least one follow-up computed tomography (CT) scan were included in this study. The patients were excluded from the final analysis if they were admitted >72 h after the aneurysmal bleeding event, and/or underwent no CT scan after a neurological deterioration. Upon receiving approval from the institutional ethics committee at the Medical Faculty of the University of Duisburg-Essen (registration number: 15-6331-BO), the observational cohort study was registered with the German clinical trial registry (DRKS) under the unique identifier DRKS00008749.

### 2.2. Subarachnoid Hemorrhage Management

All patients who were part of the study were admitted to our neurosurgical intensive care unit and received conservative treatment for SAH based on the most up-to-date guidelines [18,19]. The ruptured aneurysm was detected through digital subtraction angiography (DSA) or cranial CT angiography. Depending on the size, configuration, and location of the aneurysm, either clipping or coiling was performed within 24 h of admission. According to in-house treatment standards, the treating interventional neuroradiologist determined the indication for post-interventional antiplatelet therapy based on the risk of postprocedural thromboembolic events. Depending on the type of intervention, patients received either aspirin 100 mg as monotherapy or a combination of aspirin 100 mg and clopidogrel 75 mg (for detailed protocols on post-interventional antiplatelet therapy, please refer to our previous work [20]).

To ensure that systolic blood pressure remained under control, it was kept below 150 mmHg until the aneurysm was secured. For patients with neurologically unevaluable status and/or acute hydrocephalus, an external ventricular drainage (EVD) was inserted to enable ICP monitoring. Mean arterial pressure was maintained using colloids and/or catecholamines to ensure adequate cerebral perfusion pressure in accordance with established guidelines [18,19]. Based on in-house protocols [21], vasospasm management primarily followed a conservative approach but included interventional procedures when necessary.

### 2.3. Indications for CT Scans and Definition of Cerebral Infarction

Routine CT scans were performed on admission, within 24 h of aneurysm treatment, after placement of an EVD or any other neurosurgical intervention, and as needed based on clinical indications such as neurologic deterioration, persistent ICP increase of >20 mmHg, or impaired consciousness without any sedation. All follow-up CT scans performed within 100 days post-SAH, stored in the institution’s picture archiving and communication system, were reviewed by the first author (P.K.), who was blinded to clinical information at the time of evaluation. Cerebral infarction was defined as a new hypodense lesion on CT not attributed to intraoperative surgical manipulations or intracerebral hemorrhage (ICH), detected within the 100-day observation window.

### 2.4. Data Management

In addition to the morphological CT scan evaluation of all patients regarding the occurrence of cerebral infarction and their timing, various demographic and clinical data available at the time of aneurysm treatment were collected from the institutional SAH database. Moreover, additional patient characteristics and initial disease-related complications were collected from the entire internal hospital database. The following variables were recorded for further analyses: demographic characteristics (patients’ age, sex, and ethnicity); previous medical history and regular medication including drugs prescribed before and continued during SAH, as well as those initiated post-interventionally (e.g., aspirin); baseline SAH characteristics (presence of ICH, intraventricular hemorrhage (IVH), acute hydrocephalus and aneurysm rebleeding, original Fisher score [22], World Federation of Neurosurgical Societies (WFNS) grade [23], and treatment modality). For the subsequent statistical analyses, continuous and categorical variables were dichotomized based on standard criteria: the WFNS scale was categorized into good (1–3) and poor (4–5) grades, the Fisher score into low (1–2) and high (3–4) grades, and the modified Rankin Scale (mRS) [24] was dichotomized into favorable (1–3) versus unfavorable (4–6) outcomes.

### 2.5. Study Endpoints and Statistical Analysis

The primary objective of the study was to identify baseline patient and SAH characteristics available at the time of aneurysm treatment that were associated with 100-day infarct-free survival following SAH. Additionally, the impact of infarct timing on in-hospital mortality and unfavorable outcomes at six months post-SAH (defined as mRS > 3) was evaluated in the subgroup of patients with cerebral infarcts.

Statistical analyses were conducted using SPSS (Version 25, SPSS Inc., IBM), while GraphPad Prism 5 was used for data visualization and graphical representation. Differences with a *p*-value < 0.05 were considered statistically significant. Initially, univariate Cox regression analysis was performed for each potential predictor of infarct-free survival. Predictors found to be significant were subsequently included in a multivariable Cox regression model to identify independent predictors of post-SAH infarct-free survival. Missing data were replaced using multiple imputations in SPSS. The assumption of proportional hazards for the variables included in the Cox regression analysis was checked and confirmed by computing the time-dependent variables in SPSS.

Binary logistic regression analysis was performed to evaluate the association between infarct timing and outcome endpoints, including in-hospital mortality and unfavorable outcomes at six months. Additionally, receiver operating characteristic (ROC) curve analysis was used to determine the most clinically relevant cutoff for infarct timing in predicting SAH outcomes.

## 3. Results

### 3.1. Patient Population and Baseline Characteristics

Consecutive cases from January 2003 to June 2016 were included in our entire data collection on SAH patients. After the exclusion of cases with delayed admission and/or missing follow-up CT imaging, a total of 988 SAH patients were included in the final analysis. The main baseline and peri-interventional characteristics of the cohort are shown in Table 1.

### 3.2. Infarct Occurrence and Timing

**Figure 1:**
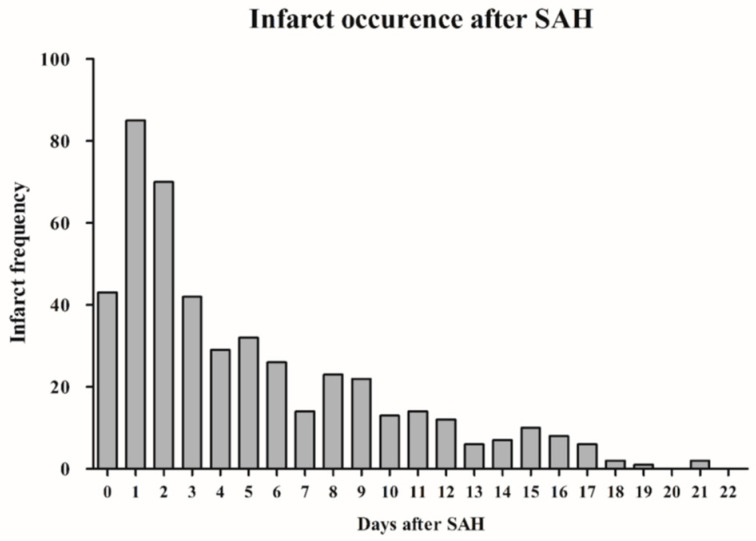
Bar chart illustrating the time course of cerebral infarctions following SAH. (The initial 22 days post-SAH are displayed for clearer visualization.) **Abbreviations**: SAH, subarachnoid hemorrhage.

Cerebral infarction during hospitalization was documented in CT scans of 475 (48.1%) patients. The median infarct occurrence time was 3.4 days post-SAH (range 0–92 days). The analysis of infarct frequency revealed that the highest daily burden of cerebral infarcts was observed on the first (8.5%) and second (7.1%) day after aneurysm hemorrhage (Figure 1), with cerebral infarction occurring in 70.9% of cases within the first 7 days. SAH individuals with cerebral infarction were at higher risk of in-hospital mortality (odds ratio [OR] = 19.15, 95% confidence interval [CI] = 10.90–33.64, *p* < 0.0001) and unfavorable outcome at 6 months after SAH (OR = 11.71, 95% CI = 8.45–16.22, *p* < 0.0001).

### 3.3. Prognostic Factors for Infarct-Free Survival

**Figure 2:**
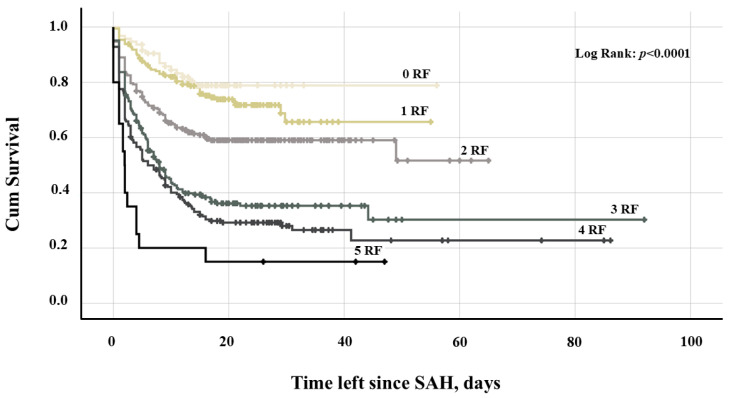
Kaplan-Meier survival plot depicting the probability of infarct-free survival after SAH in patients with 0–5 RFs: acute hydrocephalus, IVH, aneurysm rebleeding, WFNS grade ≥ 4, and absence of aspirin medication. **Abbreviation**: IVH, intraventricular hemorrhage; RF, risk factor; SAH, subarachnoid hemorrhage; WFNS, World Federation of Neurosurgical Societies.

In the univariate analysis, various demographic and clinical parameters, as well as SAH-related characteristics were first assessed for their association with infarct-free survival (Table 2). The subsequent final multivariate Cox regression analysis identified independent associations between infarct-free survival and an unfavorable initial clinical condition (WFNS ≥ 4, adjusted hazard ratio [aHR] = 1.82, 95% CI = 1.47–2.25, *p* < 0.0001), the initial presence of IVH (aHR = 1.25, 95% CI = 1.01–1.55, *p* = 0.041), aneurysm rebleeding (aHR = 1.76, 95% CI = 1.28–2.41, *p* < 0.0001), acute hydrocephalus (aHR = 1.38, 95% CI = 1.05–1.82, *p* = 0.020) and daily aspirin intake after aneurysm occlusion (aHR = 0.68, 95% CI = 0.53–0.87, *p* = 0.002, Table 3). The greater the number of unfavorable baseline characteristics (WFNS ≥ 4, IVH, aneurysm rebleeding, acute hydrocephalus, and absence of aspirin post-intervention), the higher the rate and the earlier the occurrence of cerebral infarction following SAH. Specifically, follow-up CT scans detected infarctions in 18.9%, 27%, 40.7%, 63%, 71.4%, and 85% of cases, occurring at median post-SAH days 6.8, 5.7, 3.2, 3.6, 2.8, and 1.7, respectively, in patients with zero, one, two, three, four, or five baseline risk factors (Table 4). A Kaplan-Meier survival plot illustrates the distinct trajectories of infarct-free survival in individuals with and without these baseline characteristics (Figure 2).

### 3.4. Association Between Infarct Timing and SAH Outcome

**Figure 3:**
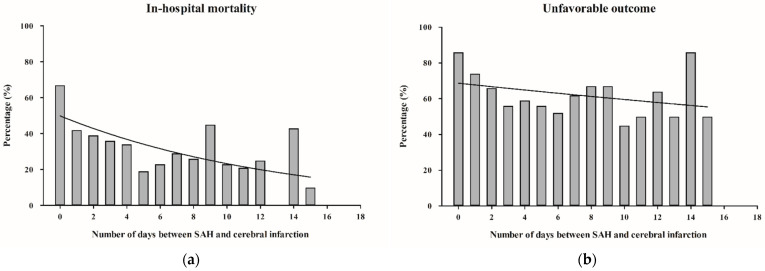
(**a**,**b**): Bar charts showing the percentage distribution of in-hospital mortality (**a**) and unfavorable outcome after 6 months (**b**) depending on the number of days between SAH and cerebral infarction. (The initial 18 days post-SAH are displayed for clearer visualization). **Abbreviations**: SAH, subarachnoid hemorrhage.

A subgroup analysis of patients with cerebral infarction showed a clear association between the timing of cerebral infarction and in-hospital mortality (OR = 0.91 per day increase, 95% CI = 0.87–0.95, *p* < 0.0001) as well as unfavorable outcome at 6 months (OR = 0.96 per day increase, 95% CI = 0.92–0.99, *p* = 0.012) after SAH. The percentage distribution and graphical representation of in-hospital mortality and unfavorable outcomes over the days following SAH showed a clear decreasing trend, whereas in-hospital mortality showed a more prominent downward trend compared to unfavorable outcomes (Figure 3a,b). According to the ROC analysis, cerebral infarction occurring within 48 h of SAH had the most significant clinical impact on SAH patients (Figure S1a,b).

## 4. Discussion

Our study provides important insights into the timing and predictors of cerebral infarction in patients with aneurysmal SAH. Analyzing a large cohort of 988 patients, we emphasize the significant impact of infarct timing on morbidity and mortality, highlighting the need for early risk recognition and targeted interventions to enable more patients to achieve infarct-free survival.

### 4.1. Infarct Timing After SAH

In our study, nearly half (48.1%) of SAH patients developed cerebral infarctions, with the majority occurring within the first week post-ictus. The median onset was 3.4 days, with 70.9% of infarctions occurring within 7 days. Notably, infarctions within the first 48 h had the most detrimental impact on outcomes, including in-hospital mortality and poor outcome at 6 months. These findings emphasize the critical nature of early brain injury (EBI) in post-SAH pathophysiology, primarily driven by increased ICP, disrupted cerebral autoregulation, and neuroinflammation [3,13,25,26]. While the exact mechanisms remain unclear, our study establishes a significant correlation between early infarction onset and adverse clinical outcomes. Infarct timing is often discussed in relation to delayed cerebral ischemia (DCI), typically occurring between days 4 and 14 [27,28], and linked to vasospasm and other secondary mechanisms [29,30,31]. Following the definitions outlined by Vergouwen et al. (2010) [5], the delayed CT-detected infarcts in our study represent DCI. Our study, however, focuses on the timing of infarction rather than on a categorization between EBI and DCI. Thus, our results provide a framework for understanding the temporal dynamics of infarction after SAH, opening a new perspective for the evaluation of SAH-related cerebral infarcts.

Reported infarct rates in SAH literature vary widely, ranging from 13% to 65% [9,10,11,12], due to differences in patient populations, methodologies, and infarct definitions. Factors such as EBI and DCI inclusion criteria, treatment protocols, frequency of CT scans, and infarct classification influence these discrepancies. Our 48.1% infarct rate aligns with the higher range of reported incidences, supporting the robustness of our findings. Several studies have investigated infarct timing beyond the acute phase. Rabinstein et al. (2005) analyzed infarct timing and distribution in SAH patients, reporting a mean interval of 12 days (range: 5–32 days) between SAH onset and the last acute hospitalization CT scan [10]. Their study delineated infarction patterns during hospitalization but lacked long-term follow-up data. Furthermore, Schmidt et al. (2022) reviewed the timeline of DCI following SAH [32]. While this study acknowledges the importance of understanding the temporal risk profile of infarction, the timing of infarction was dichotomized, categorizing infarctions as occurring either before or after day 7 after SAH. Vergouwen et al. (2011) conducted a detailed analysis of infarct timing, demonstrating that infarcts contribute to poor outcomes through both vasospasm-dependent and -independent mechanisms [33]. However, the authors appear to have classified the timeline dichotomously. Additionally, the total follow-up period was limited to six weeks. Kivisaar et al. (2001) extended follow-up to approximately 90 days, incorporating magnetic resonance imaging (MRI) alongside CT to detect infarcts [34]. They reported infarctions in 57% of patients at three months but did not specify whether infarct timing was analyzed as a continuous or dichotomous variable. Additionally, details on CT scan timing throughout the study were lacking.

While these studies provide valuable insights into long-term infarct development, they often rely on dichotomized infarct timing, limiting the ability to assess continuous infarction progression. Our study overcomes this limitation by offering a detailed, continuous infarct timing analysis, yielding a more comprehensive understanding of infarction dynamics post-SAH. The importance of long-term imaging in infarct assessment is increasingly recognized. However, a methodological gap persists in continuous temporal infarct analysis. Our findings contribute to bridging this gap by providing a time-dependent approach to infarct timing, potentially enhancing patient management and prognostic accuracy.

### 4.2. Predictors of Infarct-Free Survival

It is crucial to emphasize that our study focused on identifying early predictors of infarct-free survival based on baseline characteristics and SAH-associated factors available at the time of aneurysm occlusion. Early identification of modifiable risk factors enables timely clinical interventions, potentially reducing complications. This approach facilitates stratification of high-risk patients and supports the development of individualized treatment strategies.

Key predictors influencing infarct timing and reducing infarct-free survival included poor initial clinical condition (WFNS grade ≥ 4), IVH, aneurysm rebleeding, acute hydrocephalus, and absence of medication with aspirin post-intervention. Considering these factors could contribute to the development of more precise risk models and the establishment of evidence-based, individualized treatment algorithms aimed at improving patient outcomes after SAH.

While numerous studies have examined risk factors for infarction after aneurysmal SAH, data on predictors influencing infarct timing remain scarce.

Based on predictors that were partially included in the baseline characteristics, Jabbarli et al. (2015) developed the BEHAVIOR score [31]—an instrument for the early identification of patients at high risk for cerebral infarction following an SAH. Although the BEHAVIOR score demonstrated high diagnostic accuracy in predicting infarction risk, it does not provide information regarding the timing of the infarction and is limited to estimating the absolute risk. In a subsequent study by the same research group, the focus shifted to predicting the timing of infarctions. Only two predictors for early infarctions were identified: poor clinical condition at presentation (WFNS grade ≥ 4) and the presence of IVH [9]. The WFNS scale is a well-established, non-modifiable predictor of aneurysmal SAH severity and prognosis. While it cannot be altered, intensive monitoring in high-risk patients can improve outcomes by enabling early detection and management of secondary complications such as vasospasm, elevated ICP, and hydrocephalus. Evidence supports the benefits of close surveillance, with studies showing that timely interventions enhance recovery and reduce long-term deficits, while tailored monitoring strategies are emphasized to optimize patient-specific risk management and prognosis [35,36,37]. These findings underscore the critical role of proactive monitoring in mitigating secondary complications and improving clinical outcomes.

Aneurysm rebleeding before interventional or surgical treatment is a severe and well-documented complication of SAH, often resulting in worsened neurological outcomes and increased mortality. Recognizing rebleeding as a key predictor of infarction timing raises the critical question of whether it can be prevented and, if so, what strategies are most effective. Darkwah Oppong et al. (2019) identified several risk factors that significantly increase the likelihood of rebleeding prior to treatment [38]. They emphasized the importance of proactive monitoring and early intervention in reducing the risk of rebleeding and improving patient outcomes.

IVH and acute hydrocephalus, both identified as predictors in our study, are increasingly recognized as modifiable factors. Given their frequent causal relationship, addressing one condition can often mitigate the effects of the other. Larger IVH volumes are associated with poorer outcomes due to elevated ICP and impaired cerebrospinal fluid circulation [39] often caused by ventricular obstruction. These factors have fueled growing interest in therapeutic strategies to reduce the impact of both IVH and acute hydrocephalus. Intraventricular thrombolytic therapy has gained attention as a potential intervention, with evidence suggesting it can reduce clot burden and improve recovery [40,41]. Meta-analyses suggest that cisternal and intraventricular irrigation accelerates hematoma resolution and enhances functional outcomes [40]. In addition to intraventricular thrombolytic therapy, early placement of lumbar drainage in patients with aneurysmal SAH has been associated with improved clinical outcomes. Studies have shown that early lumbar drainage can lead to a reduction in secondary infarctions and mitigate complications such as vasospasm. These benefits are primarily attributed to the drainage of blood and cerebrospinal fluid, which lowers intracranial pressure and enhances cerebral perfusion. Acute hydrocephalus, whether resulting from IVH or not, is a key predictor of cerebral infarction in patients with aneurysmal SAH. It increases the risk of infarction by a factor of 6.67 [42] and is strongly associated with a higher likelihood of DCI, highlighting its critical role in infarct development [43]. These findings highlight the importance of early detection and management of hydrocephalus and IVH to reduce the risk of infarction and improve outcomes [44,45]. Timely interventions, such as EVD placement, potentially in combination with lumbar drainage, can mitigate infarction [44,45]. Based on these observations, acute hydrocephalus and IVH appear to be both early predictors and modifiable risk factors. Therefore, targeted therapies for both factors should be prioritized to improve long-term recovery and prognosis.

Our findings also suggest a potential protective role of aspirin in infarction risk reduction. Cagnazzo et al. (2019) reported that while antiplatelet therapy did not significantly decrease overall DCI incidence, patients on long-term aspirin therapy post-endovascular treatment had lower DCI rates and improved outcomes [46]. Despite these promising results, the routine use of aspirin or other antiplatelet agents remains controversial due to concerns about hemorrhagic complications. For instance, van den Bergh et al. (2009) reported that antiplatelet therapy did not significantly improve outcomes in SAH patients and may even have increased the risk of bleeding [47]. However, more recent studies, such as Darkwah Oppong et al. (2020), reported that while antiplatelet therapy, including aspirin, did increase minor bleeding, these events were clinically insignificant [48]. In contrast, major bleeding, which is linked to worse neurological outcomes, was significantly associated with anticoagulation. Furthermore, Engel et al. (2024) provided evidence supporting the potential benefits of aspirin in SAH patients [49]. Their study revealed that severe carotid siphon calcification negatively impacted functional outcomes only in patients not receiving aspirin. These findings suggest that aspirin may offer neuroprotective effects in specific subgroups, highlighting the need for further research into its role in SAH treatment.

## 5. Conclusions

Our study provides valuable insights into the timing and predictors of cerebral infarction in aneurysmal SAH. We found that nearly half of SAH patients experience infarction, with the majority occurring within the first week post-ictus. Early infarcts, particularly within 48 h, were associated with the poorest outcomes. Identifying key predictors—including poor initial clinical condition, intraventricular hemorrhage, aneurysm rebleeding, acute hydrocephalus, and the absence of aspirin therapy—allows for early risk stratification and targeted interventions. Notably, our approach of analyzing infarction as a continuous variable enables a more nuanced understanding of its progression, addressing gaps left by prior dichotomized classifications. These findings underscore the need for early and individualized treatment strategies. Future research should focus on refining risk models and evaluating targeted therapeutic interventions to improve patient outcomes in SAH management.

## 6. Study Limitations

This study has several limitations that should be considered when interpreting the findings. As a single-center retrospective analysis, the results may not be generalizable to other populations or healthcare settings. Variability in management protocols and patient demographics could have influenced outcomes.

Furthermore, the reliance on CT imaging for the detection of cerebral infarctions likely underestimates the true incidence, particularly of microinfarctions, which are more accurately identified using advanced modalities such as diffusion-weighted MRI. This limitation may have implications for the assessment of the full spectrum of infarct burden in SAH patients. We also acknowledge that patients who died very shortly after admission did not have the opportunity to receive aspirin. Future multicenter, prospective studies with more comprehensive imaging protocols are needed to validate these findings and further refine our understanding of infarction dynamics in SAH.

## Figures and Tables

**Table 1:** Major baseline characteristics of the patients in the final cohort.

Demographic Characteristics:	n (% *) or Mean (±SD)
Number of patients	988
Age, years	54.7 (±13.9)
Sex, female	666 (67.4%)
SAH characteristics:	
WFNS, grade 4–5	413 (41.8%)
Fisher, grade 3–4	753 (76.2%)
Presence of ICH	303 (30.7%)
Presence of IVH	451 (45.6%)
Aneurysm rebleeding	58 (5.9%)
Treatment modality, clipping	365 (36.9%)
Comorbidities:	
Arterial hypertension	682 (69.0%)
Hypercholesterinemia	80 (8.1%)
Hypothyroidism	109 (11.0%)
Hyperthyroidism	11 (1.1%)
Hyperuricemia	25 (2.5%)
Cardiac comorbidity	104 (10.5%)
Diabetes mellitus	53 (5.4%)
Renal comorbidity	36 (3.6%)
Liver comorbidity	31 (3.1%)
Pulmonary comorbidity	79 (8.0%)
Peripheral arterial disease	14 (1.4%)
Medication:	
Beta blockers (regular medication)	147 (14.9%)
Chronic use of anti-inflammatory drugs (non-steroidal/steroidal)	65 (6.6%)
Calcium channel blockers (regular medication)	93 (9%)
ACE inhibitors (regular medication)	169 (17%)
AT1 receptor blockers (regular medication)	55 (5.6%)
Statins (regular medication)	52 (5.2%)
Aspirin (after aneurysm occlusion)	333 (4%)

Abbreviations: ACE, angiotensin converting enzyme; AT1, angiotensin 1; ICH, intracerebral hemorrhage; IVH, intraventricular hemorrhage; SD, standard deviation; SAH, subarachnoid hemorrhage; WFNS, World Federation of Neurosurgical Societies. *—percentages were calculated based on the cases with known values.

**Table 2:** Univariate Cox regression analysis of the predictors of infarct-free survival.

Dichotomous Variables:	Infarct-Free Survival	
	HR (95% CI)	*p*-Value
Demographic characteristics:		
Age (per year increase)	1.01 (1.00–1.01)	0.014
Sex (female)	0.95 (0.79–1.15)	0.60
Ethnicity (non-Caucasian)	1.46 (0.99–2.15)	0.06
SAH characteristics:		
Fisher grade 3–4	2.19 (1.55–3.09)	<0.0001
Presence of IVH	1.95 (1.63–2.35)	<0.0001
Presence of ICH	1.49 (1.24–1.80)	<0.0001
WFNS grade 4–5	2.49 (2.07–2.98)	<0.0001
Treatment modality (clipping)	1.41 (1.17–1.71)	0.0004
Aneurysm rebleeding	2.48 (1.83–3.37)	<0.0001
Acute hydrocephalus	2.16 (1.71–2.72)	<0.0001
Comorbidities:		
Arterial hypertension	1.11 (0.91–1.35)	0.32
Hypothyroidism	0.79 (0.58–1.08)	0.15
Hyperthyroidism	0.71 (0.26–1.89)	0.49
Hyperuricemia	1.24 (0.72–2.16)	0.44
Cardiac comorbidity	1.46 (1.12–1.91)	0.006
Diabetes mellitus	0.85 (0.56–1.31)	0.47
Renal comorbidity	1.11 (0.68–1.80)	0.68
Liver comorbidity	0.72 (0.40–1.32)	0.29
Pulmonary comorbidity	0.85 (0.59–1.21)	0.35
Peripheral arterial disease	1.14 (0.54–2.41)	0.73
Medication:		
Beta-blockers (regular medication)	1.15 (0.90–1.48)	0.27
Chronic use of anti-inflammatory drugs (non-steroidal/steroidal)	0.63 (0.41–0.97)	0.037
Calcium channel blockers (regular medication)	1.44 (1.09–1.90)	0.010
ACE inhibitors (regular medication)	0.89 (0.70–1.14)	0.36
AT1 receptor blockers (regular medication)	1.15 (0.79–1.67)	0.47
Statins (regular medication)	1.15 (0.78–1.69)	0.49
Aspirin (after aneurysm occlusion)	0.57 (0.46–0.70)	<0.0001

Abbreviations: ACE, angiotensin converting enzyme; AT1, angiotensin 1; CI, confidence interval; HR, hazard ratio; ICH, intracerebral hemorrhage; IVH, intraventricular hemorrhage; SAH, subarachnoid hemorrhage; WFNS, World Federation of Neurosurgical Societies.

**Table 3:** Multivariate analysis of the predictors of infarct-free survival.

Predictors of Infarct-Free Survival	aHR (95% CI)	*p*-Value
Age (per year increase)	1.00 (0.99–1.01)	0.88
WFNS (grade 4–5)	1.82 (1.47–2.25)	<0.0001
Fisher (grade 3–4)	1.21 (0.83–1.77)	0.33
Presence of IVH	1.25 (1.01–1.55)	0.04
Presence of ICH	0.95 (0.77–1.18)	0.64
Acute hydrocephalus	1.38 (1.05–1.82)	0.02
Cardiac comorbidity	1.18 (0.90–1.57)	0.23
Chronic use of anti-inflammatory drugs (non-steroidal/steroidal)	0.70 (0.46–1.08)	0.11
Medication with calcium channel blockers	1.24 (0.94–1.64)	0.12
Medication with aspirin	0.68 (0.53–0.87)	0.002
Treatment modality (clipping)	1.16 (0.93–1.44)	0.19
Aneurysm rebleeding	1.76 (1.28–2.41)	0.0004

Abbreviations: aHR, adjusted hazard ratio; CI, confidence interval; ICH, intracerebral hemorrhage; IVH, intraventricular hemorrhage; WFNS, World Federation of Neurosurgical Societies.

**Table 4:** Presentation of the absolute (n) and percentage proportions of cerebral infarctions and the median day of infarct timing in the presence of 0 to 5 RFs (acute hydrocephalus, IVH, aneurysm rebleeding, WFNS grade ≥ 4, and absence of aspirin medication) after SAH.

*n* of RF	Infarct Occurrence	Infarct Timing in Days
	*n* of SAH-Cases (%)	Median (IQR: 25–75%)
0	18 (18.9)	6.8 (3–10)
1	53 (27.0)	5.7 (3–12)
2	100 (40.7)	3.2 (1–8)
3	143 (63.0)	3.6 (1–7)
4	140 (71.4)	2.8 (1–8)
5	17 (85.0)	1.7 (1–2)
1–5	453 (51.2)	3.3 (1–8)

**Abbreviations:** IQR, interquartile range; IVH, intraventricular hemorrhage; RF, risk factor; SAH, subarachnoid hemorrhage; WFNS, World Federation of Neurosurgical Societies.

## Data Availability

The data presented in this study are not publicly available due to institutional data protection policies but are available from the corresponding author upon reasonable request.

## Supplementary Materials

The following supporting information can be downloaded at: https://www.mdpi.com/article/10.3390/brainsci15101042/s1, Figure S1: ROC curve analysis of clinically relevant infarct timing for the study endpoints.

## References

[1] de Oliveira Manoel A.L., Mansur A., Silva G.S., Germans M.R., Jaja B.N.R., Kouzmina E., Marotta T.R., Abrahamson S., Schweizer T.A., Spears J. (2016). Functional Outcome After Poor-Grade Subarachnoid Hemorrhage: A Single-Center Study and Systematic Literature Review. Neurocritical Care.

[2] Nieuwkamp D.J., E Setz L., Algra A., Linn F.H., de Rooij N.K., Rinkel G.J. (2009). Changes in case fatality of aneurysmal subarachnoid haemorrhage over time, according to age, sex, and region: A meta-analysis. Lancet Neurol..

[3] Alsbrook D.L., Di Napoli M., Bhatia K., Desai M., Hinduja A., Rubinos C.A., Mansueto G., Singh P., Domeniconi G.G., Ikram A. (2023). Pathophysiology of Early Brain Injury and Its Association with Delayed Cerebral Ischemia in Aneurysmal Subarachnoid Hemorrhage: A Review of Current Literature. J. Clin. Med..

[4] Springer M.V., Schmidt J.M., Wartenberg K.E., Frontera J.A., Badjatia N., Mayer S.A. (2009). Predictors of global cognitive impairment 1 year after subarachnoid hemorrhage. Neurosurgery.

[5] Vergouwen M.D., Vermeulen M., Van Gijn J., Rinkel G.J., Wijdicks E.F., Muizelaar J.P., Mendelow A.D., Juvela S., Yonas H., Terbrugge K.G. (2010). Definition of delayed cerebral ischemia after aneurysmal subarachnoid hemorrhage as an outcome event in clinical trials and observational studies: Proposal of a multidisciplinary research group. Stroke.

[6] Brami J., Chousterman B., Boulouis G., Le Dorze M., Majlath M., Saint-Maurice J.-P., Civelli V., Froelich S., Houdart E., Labeyrie M.-A. (2020). Delayed cerebral infarction is systematically associated with a cerebral vasospasm of large intracranial arteries. Neurosurgery.

[7] Macdonald R.L. (2014). Delayed neurological deterioration after subarachnoid haemorrhage. Nat. Rev. Neurol..

[8] Geraghty J.R., Testai F.D. (2017). Delayed Cerebral Ischemia after Subarachnoid Hemorrhage: Beyond Vasospasm and Towards a Multifactorial Pathophysiology. Curr. Atheroscler. Rep..

[9] Jabbarli R., Reinhard M., Niesen W., Roelz R., Shah M., Kaier K., Hippchen B., Taschner C., Van Velthoven V. (2015). Predictors and impact of early cerebral infarction after aneurysmal subarachnoid hemorrhage. Eur. J. Neurol..

[10] Rabinstein A.A., Weigand S., Atkinson J.L., Wijdicks E.F. (2005). Patterns of cerebral infarction in aneurysmal subarachnoid hemorrhage. Stroke.

[11] Qureshi A.I., Bhatti I.A., Gillani S.A., Beall J., Cassarly C.N., Gajewski B., Martin R.H., Suarez J.I., Kwok C.S. (2024). Prevalence, trends, and outcomes of cerebral infarction in patients with aneurysmal subarachnoid hemorrhage in the USA. J. Neuroimaging.

[12] Veldeman M., Rossmann T., Haeren R., Vossen L.V., Weiss M., Conzen C., Siironen J.O., Korja M., Schmidt T.P., Höllig A. (2024). Delayed Cerebral Infarction After Aneurysmal Subarachnoid Hemorrhage: Location, Distribution Patterns, Infarct Load, and Effect on Outcome. Neurology.

[13] Li X., Zeng L., Lu X., Chen K., Yu M., Wang B., Zhao M. (2023). Early Brain Injury and Neuroprotective Treatment after Aneurysmal Subarachnoid Hemorrhage: A Literature Review. Brain Sci..

[14] Ayling O.G.S., Ibrahim G.M., Alotaibi N.M., Gooderham P.A., Macdonald R.L. (2016). Dissociation of early and delayed cerebral infarction after aneurysmal subarachnoid hemorrhage. Stroke.

[15] Rabinstein A.A., Friedman J.A., Weigand S.D., McClelland R.L., Fulgham J.R., Manno E.M., Atkinson J.L., Wijdicks E.F. (2004). Predictors of cerebral infarction in aneurysmal subarachnoid hemorrhage. Stroke.

[16] Schmidt J.M., Rincon F., Fernandez A., Resor C., Kowalski R.G., Claassen J., Connolly E.S., Fitzsimmons B.-F.M., Mayer S.A. (2007). Cerebral infarction associated with acute subarachnoid hemorrhage. Neurocritical Care.

[17] Lee V.H., Ouyang B., John S., Conners J.J., Garg R., Bleck T.P., Temes R.E., Cutting S., Prabhakaran S. (2014). Risk stratification for the in-hospital mortality in subarachnoid hemorrhage: The HAIR score. Neurocritical Care.

[18] Bederson J.B., Connolly E.S., Batjer H.H., Dacey R.G., Dion J.E., Diringer M.N., Duldner J.E., Harbaugh R.E., Patel A.B., Rosenwasser R.H. (2009). Guidelines for the management of aneurysmal subarachnoid hemorrhage: A statement for healthcare professionals from a special writing group of the Stroke Council, American Heart Association. Stroke.

[19] Steiner T., Juvela S., Unterberg A., Jung C., Forsting M., Rinkel G., European Stroke Organization (2013). European Stroke Organization guidelines for the management of intracranial aneurysms and subarachnoid haemorrhage. Cerebrovasc Dis..

[20] Darkwah Oppong M., Gembruch O., Pierscianek D., Köhrmann M., Kleinschnitz C., Deuschl C., Mönninghoff C., Kaier K., Forsting M., Sure U. (2019). Post-treatment Antiplatelet Therapy Reduces Risk for Delayed Cerebral Ischemia due to Aneurysmal Subarachnoid Hemorrhage. Neurosurgery.

[21] Ketelauri P., Gümüs M., Gull H.H., Rieß C., Dinger T.F., Li Y., Rauschenbach L., Ahmadipour Y., Oppong M.D., Dammann P. (2025). The course and clinical relevance of interleukin-6 in cerebrospinal fluid in patients with aneurysmal subarachnoid hemorrhage. World Neurosurg..

[22] Fisher C.M., Roberson G.H., Ojemann R.G. (1977). Cerebral vasospasm with ruptured saccular aneurysm—The clinical manifestations. Neurosurgery.

[23] Teasdale G.M., Drake C.G., Hunt W., Kassell N., Sano K., Pertuiset B., De Villiers J.C. (1988). A universal subarachnoid hemorrhage scale: Report of a committee of the World Federation of Neurosurgical Societies. J. Neurol. Neurosurg. Psychiatry.

[24] van Swieten J.C., Koudstaal P.J., Visser M.C., Schouten H.J., van Gijn J. (1988). Interobserver agreement for the assessment of handicap in stroke patients. Stroke.

[25] Ikram A., Javaid M.A., Ortega-Gutierrez S., Selim M., Kelangi S., Anwar S.M.H., Torbey M.T., Divani A.A. (2021). Delayed Cerebral Ischemia after Subarachnoid Hemorrhage. J. Stroke Cerebrovasc. Dis..

[26] Frontera J.A., Provencio J.J., Sehba F.A., McIntyre T.M., Nowacki A.S., Gordon E., Weimer J.M., Aledort L. (2017). The Role of Platelet Activation and Inflammation in Early Brain Injury Following Subarachnoid Hemorrhage. Neurocritical Care.

[27] Dorsch N.W., King M.T. (1994). A review of cerebral vasospasm in aneurysmal subarachnoid haemorrhage Part I: Incidence and effects. J. Clin. Neurosci..

[28] Rowland M.J., Hadjipavlou G., Kelly M., Westbrook J., Pattinson K. (2012). Delayed cerebral ischaemia after subarachnoid haemorrhage: Looking beyond vasospasm. BJA Br. J. Anaesth..

[29] Jabbarli R., Pierscianek D., Oppong M.D., Sato T., Dammann P., Wrede K.H., Kaier K., Köhrmann M., Forsting M., Kleinschnitz C. (2020). Laboratory biomarkers of delayed cerebral ischemia after subarachnoid hemorrhage: A systematic review. Neurosurg. Rev..

[30] de Oliveira Manoel A.L., Jaja B.N., Germans M.R., Yan H., Qian W., Kouzmina E., Marotta T.R., Turkel-Parrella D., Schweizer T.A., Macdonald R.L. (2015). The VASOGRADE: A Simple Grading Scale for Prediction of Delayed Cerebral Ischemia After Subarachnoid Hemorrhage. Stroke.

[31] Jabbarli R., Reinhard M., Roelz R., Shah M., Niesen W.-D., Kaier K., Taschner C., Weyerbrock A., Van Velthoven V. (2015). Early identification of individuals at high risk for cerebral infarction after aneurysmal subarachnoid hemorrhage: The BEHAVIOR score. J. Cereb. Blood Flow. Metab..

[32] Schmidt T.P., Weiss M., Hoellig A., Nikoubashman O., Schulze-Steinen H., Albanna W., Clusmann H., Schubert G.A., Veldeman M. (2022). Revisiting the Timeline of Delayed Cerebral Ischemia After Aneurysmal Subarachnoid Hemorrhage: Toward a Temporal Risk Profile. Neurocritical Care.

[33] Vergouwen M.D.I., Ilodigwe D., Macdonald R.L. (2011). Cerebral Infarction After Subarachnoid Hemorrhage Contributes to Poor Outcome by Vasospasm-Dependent and -Independent Effects. Stroke.

[34] Kivisaari R.P., Salonen O., Servo A., Autti T., Hernesniemi J., Öhman J. (2001). MR imaging after aneurysmal subarachnoid hemorrhage and surgery: A long-term follow-up study. AJNR Am. J. Neuroradiol..

[35] Robba C., Busl K.M., Claassen J., Diringer M.N., Helbok R., Park S., Rabinstein A., Treggiari M., Vergouwen M.D.I., Citerio G. (2024). Contemporary management of aneurysmal subarachnoid haemorrhage. An update for the intensivist. Intensive Care Med..

[36] Addis A., Baggiani M., Citerio G. (2023). Intracranial Pressure Monitoring and Management in Aneurysmal Subarachnoid Hemorrhage. Neurocritical Care.

[37] Hoh B.L., Ko N.U., Amin-Hanjani S., Chou S.H.-Y., Cruz-Flores S., Dangayach N.S., Derdeyn C.P., Du R., Hänggi D., Hetts S.W. (2023). 2023 Guideline for the Management of Patients with Aneurysmal Subarachnoid Hemorrhage: A Guideline from the American Heart Association/American Stroke Association. Stroke.

[38] Darkwah Oppong M., Gümüs M., Pierscianek D., Herten A., Kneist A., Wrede K., Barthel L., Forsting M., Sure U., Jabbarli R. (2019). Aneurysm rebleeding before therapy: A predictable disaster?. J. Neurosurg..

[39] Park H.G., Kim S., Chung J., Jang C.K., Park K.Y., Lee J.W. (2021). Intraventricular hemorrhage clot clearance rate as an outcome predictor in patients with aneurysmal subarachnoid hemorrhage: A retrospective study. BMC Neurol..

[40] Lind A.N.R., Krabbenhøft M.G., Valentin J.B., Haldrup M., Dyrskog S., Rasmussen M., Simonsen C.Z., Korshoej A.R. (2024). Cisternal and intraventricular irrigation in subarachnoid and intraventricular haemorrhage. Stroke Vasc. Neurol..

[41] Witsch J., Roh D.J., Avadhani R., Merkler A.E., Kamel H., Awad I., Hanley D.F., Ziai W.C., Murthy S.B. (2021). Association Between Intraventricular Alteplase Use and Parenchymal Hematoma Volume in Patients with Spontaneous Intracerebral Hemorrhage and Intraventricular Hemorrhage. JAMA Netw. Open.

[42] Fu C., Yu W., Sun L., Li D., Zhao C. (2013). Early cerebral infarction following aneurysmal subarachnoid hemorrhage: Frequency, risk factors, patterns, and prognosis. Curr. Neurovasc. Res..

[43] Parasram M., Park J., Al-Dulaimi M.W., Magid-Bernstein J.R. (2023). Guidelines in Action: Management of Acute and Chronic Hydrocephalus Following Aneurysmal Subarachnoid Hemorrhage. Stroke.

[44] Wolf S., Mielke D., Barner C., Malinova V., Kerz T., Wostrack M., Czorlich P., Salih F., Engel D.C., Ehlert A. (2023). Effectiveness of Lumbar Cerebrospinal Fluid Drain Among Patients with Aneurysmal Subarachnoid Hemorrhage: A Randomized Clinical Trial. JAMA Neurol..

[45] Al-Tamimi Y.Z., Bhargava D., Feltbower R.G., Hall G., Goddard A.J., Quinn A.C., Ross S.A. (2012). Lumbar Drainage of Cerebrospinal Fluid After Aneurysmal Subarachnoid Hemorrhage. Stroke.

[46] Cagnazzo F., Derraz I., Lefevre P.-H., Gascou G., Dargazanli C., Riquelme C., Perrini P., di Carlo D., Bonafe A., Costalat V. (2019). Antiplatelet Therapy in Patients with Aneurysmal SAH: Impact on Delayed Cerebral Ischemia and Clinical Outcome. A Meta-Analysis. Am. J. Neuroradiol..

[47] van den Bergh W.M., Kerr R.S., Algra A., Rinkel G.J., Molyneux A.J. (2009). Effect of Antiplatelet Therapy for Endovascular Coiling in Aneurysmal Subarachnoid Hemorrhage. Stroke.

[48] Darkwah Oppong M., Buffen K., Pierscianek D., Herten A., Ahmadipour Y., Dammann P., Rauschenbach L., Forsting M., Sure U., Jabbarli R. (2020). Secondary hemorrhagic complications in aneurysmal subarachnoid hemorrhage: When the impact hits hard. J. Neurosurg..

[49] Engel A., Song L., Rauschenbach L., Gümüs M., Santos A.N., Dinger T.F., Oppong M.D., Li Y., Gembruch O., Ahmadipour Y. (2024). Impact of Carotid Siphon Calcification on the Course and Outcome of Patients with Aneurysmal Subarachnoid Hemorrhage. Stroke.

