# Supplementary material for: Infarct Timing and Predictors of Infarct-Free Survival in Patients with Aneurysmal Subarachnoid Hemorrhage
Source: Brain Sci. 2025 Sep 25;15(10):1042. doi: 10.3390/brainsci15101042 (PMC12563843; doi:10.3390/brainsci15101042)

ONLINE SUPPLEMENTS

Figure S1 a, b: ROC curve analysis of clinically relevant infarct timing for the study endpoints.

a **Unfavorable outcome after 6 months**

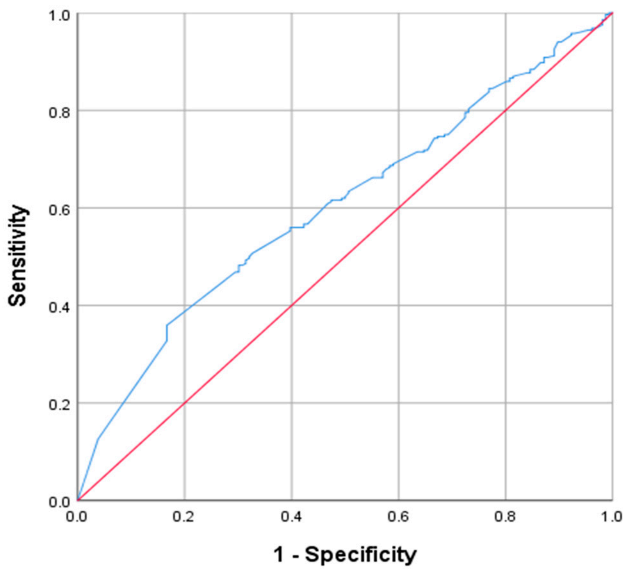

b **In-hospital mortality**

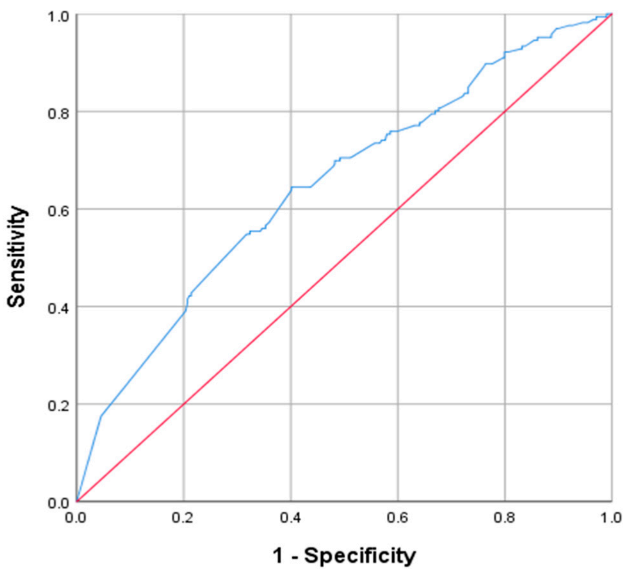

Supplement: Supplementary file 1 [file brainsci-15-01042-s001.zip › brainsci-3841871-supplementary.pdf]
